# Supplementary material for: The Rpd3-Complex Regulates Expression of Multiple Cell Surface Recycling Factors in Yeast
Source: Int J Mol Sci. 2021 Nov 19;22(22):12477. doi: 10.3390/ijms222212477 (PMC8617818; doi:10.3390/ijms222212477)
Supplement: Supplementary file 1 [file ijms-22-12477-s001.zip › ijms-1453361-supplementary/Supplemental Figures_legends_movie_tables/Supplemental Figures.pdf]

	transformant		
	1	2	3
Vector			
YAR029W			
TPS1			
VPS51			
DUR3			
PPA1			
EMP46			
PDA1			
VAM6			
ERG2			
VPS30			

	transformant		
	1	2	3
FIS1			
VNX1			
NHX1			
VPS55			
TLG2			
LTV1			
YPT11			
YPT31			
NEM1			
ERG24			
GTR1			

Supplemental Figure S1: over-expressor screen in wild-type background

Wild-type cells stably expressing Ste3-GFP-DUb were transformed with over-expression plasmids for listed genes before growth in selective media to mid-log phase and confocal microscopy. Three individual clones of each transformation was visualised but none of these over-expression plasmids induced a recycling defect of Ste3-GFP-DUb.

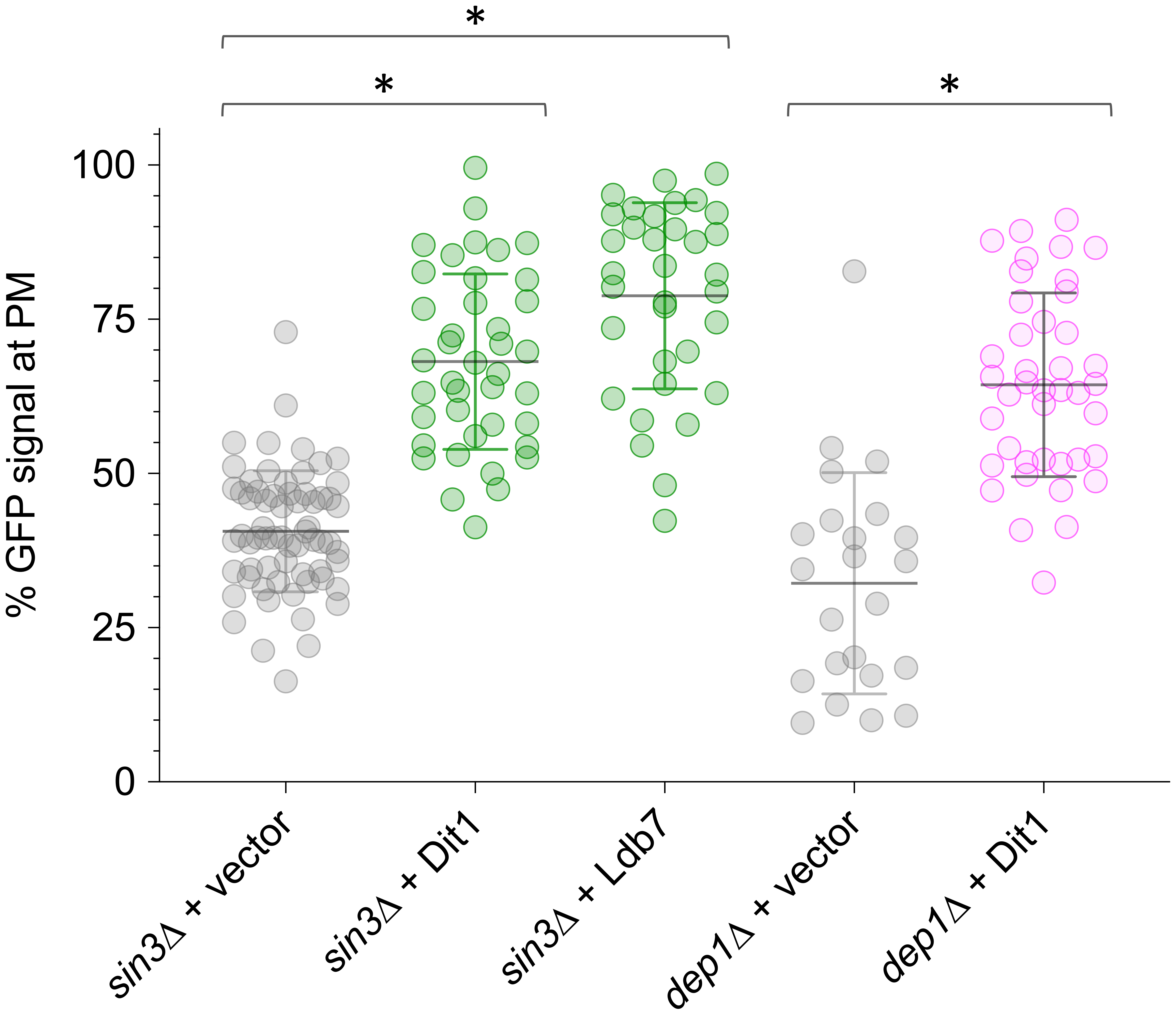

Supplemental Figure S2: Additional recycling complementation experiments

sin3Δ and *dep1Δ* mutant cells expressing Ste3-GFP-DUb from a chromosomal copy and transformed with indicated plasmids were grown to mid-log phase before confocal microscopy to determine localisation. The surface levels of Ste3-GFP-DUb in each condition was quantified as a percentage and asterisks (*) used to indicate significant difference of $p < 0.0001$ from *t*-test comparisons.

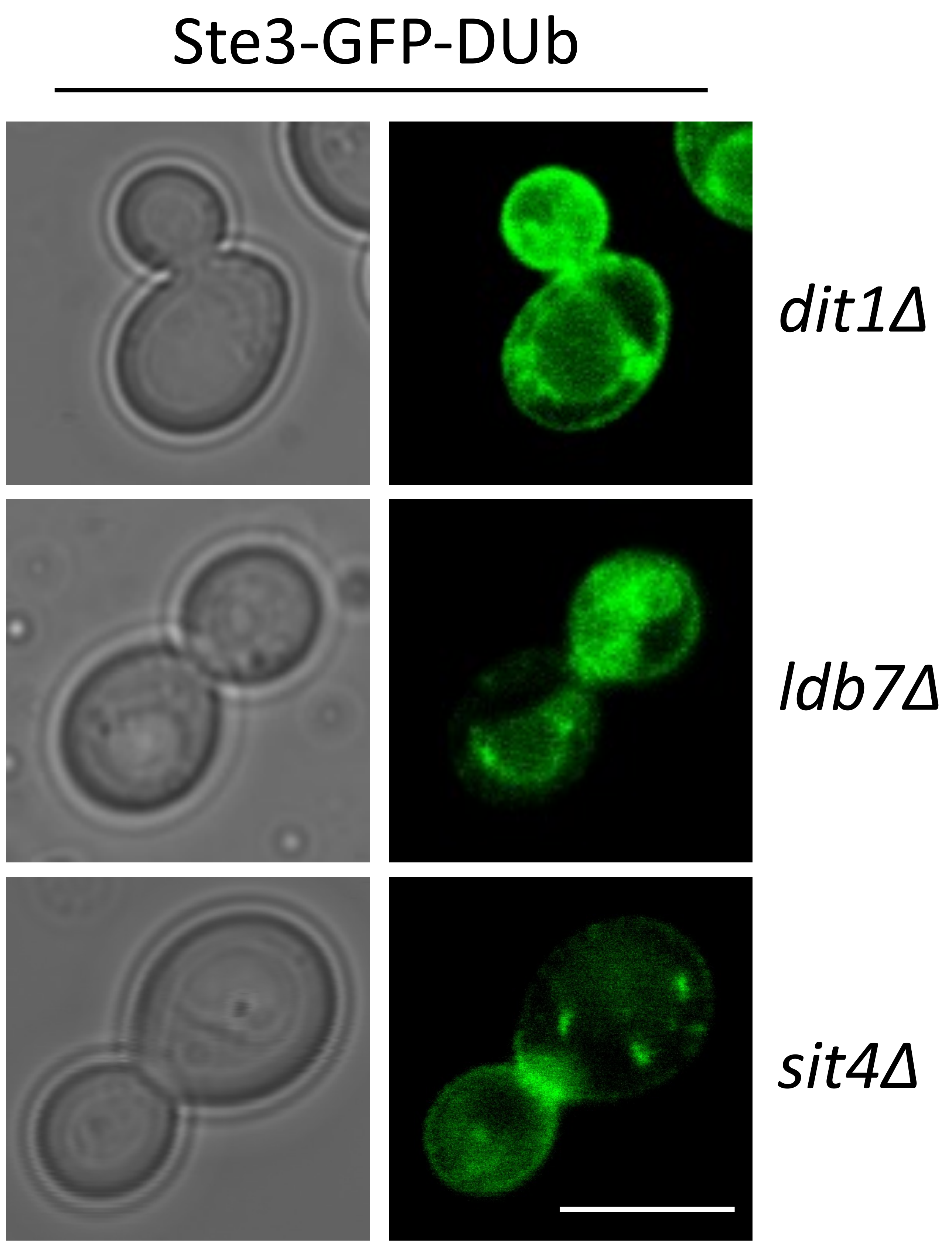

Supplemental Figure S3: Recycling defects in mutants from screen

Indicated mutants expressing Ste3-GFP-DUb were grown to mid-log phase before preparation for confocal microscopy and visualisation of the reporter. Scale bar, 5 μm.
